# Financial toxicity in cancer palliative care in India: Addressing existence and beyond – Seeking remedies for a balanced financial journey

**DOI:** 10.3332/ecancer.2024.1820

**Published:** 2024-12-12

**Authors:** Saurabh Joshi, Anuja Damani, Anant Garg, Sunny Malik, Ajay Kumar Dewan, Rakesh Sharma, Upkar Joshi

**Affiliations:** 1 Hospice Education India LLP, New Delhi 110088, India; 2 Department of Palliative Medicine and Supportive Care, Kasturba Medical College, Manipal Academy of Higher Education, Manipal 576104, India; 3 Arthroplasty and Arthroscopy SIOR, Pune, India; 4 Spinal Surgery, KUMC Seoul, Seoul, South Korea; 5 RG Kar Medical College and Hospital, Kolkata 700004, India; 6 Department of Pain Management, Hospice Care & Palliative Medicine, Rajiv Gandhi Cancer Institute and Research Centre (RGCI&RC), Niti Bagh, New Delhi 110049, India; 7 Rajiv Gandhi Cancer Institute, Sector-5, Rohini, Delhi 110085, India; 8 Rajiv Gandhi Cancer Institute, Mahender Kumar Jain Marg, Niti Bagh, New Delhi 110049, India; 9 National Board in Critical Care Medicine (DrNB-CCM), National Board of Examinations (NBE), New Delhi 110029, India; 10 West Bengal University of Health Sciences, Kolkata 700064, India; 11 Narayana Health-Rabindranath Tagore International Institute of Cardiac Sciences, Kolkata 700099, India; 12 Ascent Capital Financial Services Private Limited, New Delhi 110088, India; 13 Gargi College, University of Delhi, New Delhi 110049, India; 14 Business Economics, Bhim Rao Ambedkar College, University of Delhi, New Delhi 110032, India; 15 Academic Advisory Council, New Delhi Institute of Management, New Delhi 110062, India

**Keywords:** financial toxicity, cancer care, palliative care, financial distress, catastrophic healthcare expenditure, financial literacy, health insurance, financial planning, palliative care, transitioning, hospice care

## Abstract

Financial toxicity (FT) places a significant burden on individuals undergoing cancer care, leading to emotional distress, social isolation and financial burnout. In India, the growing number of cancer cases and the ever-expanding population, combined with insufficient government investment in public healthcare, inadequate insurance coverage, poor financial literacy among medical and non-medical communities and the lack of comprehensive financial planning exacerbate the financial difficulties faced by patients. This article aims to address FT as a source of suffering and explores potential frameworks and solutions for preventing and managing FT in patients undergoing cancer treatment and seeking palliative and hospice care in India. We conducted a literature search to review the burden of FT, across diverse healthcare settings for cancer patients. The prevalence of FT in cancer care ranges from 30% to 90.1% and is influenced by various socio-demographic, disease and healthcare-related factors. The sources of distress financing include consumption of savings, asset sales and borrowing, which add to the financial suffering. This interdisciplinary collaborative research paper highlights the dearth of financial literacy in our population and emphasises the pressing need to enhance financial awareness for healthcare professionals, cancer patients and their families. More than 30% of the Indian population lacks any form of insurance coverage, and many of those who do have it mostly lack 'adequate' coverage. We explore essential financial strategies, such as budgeting, expense analysis, asset consolidation, liquidity management, understanding estate planning tools and banking operations, streamlining paperwork, ensuring smooth transactions, adopting methods like low-interest loans and crowdfunding platforms, advance care planning, early palliative care ntegration and timely transition to hospice care. We also highlight the importance of available community resources, non-profit organisations, cancer foundations, health insurance and government support. Overall, integrating financial planning into cancer palliative care seems essential for reducing FT and enhancing the quality of cancer care in India. Further research on the topic is needed.

## Introduction and problem statement

*‘…because it’s a question of survival, problems of finance, social, emotional…all the things are attached with it…and if you were the bread earner of the family…as I am in my case, you see that particular disease in everybody’s eyes in the family. Every moment you meet your mother, your wife, your children, you can see cancer in their eyes (cries). It’s a living disease. It does not stick to my cheek or my mouth. I can feel it in my family. I can feel it in my friends. I can feel it in my circle and then you become an object of (cries)…you know…where people say, alas, why you? you are such a good man…so where ever you go, you can feel the disease sitting there. It’s not limited to your body, you have to live with it…it becomes a living partner (cries)’* [1]*.*

*‘…starting from the pre admission space, diagnosis, biopsy, the Magnetic Resonance Imaging, the Positron Emission Tomography scans, the tests and the surgery and radiation together, I think I spend around 14 lacs…14 and half, maybe 15 lacs…you know…and the process…still on. 15,000 rupees per month on my medications, tests, investigation which are going to happen in coming 6–8 months, minimum. So, the overall expense, I think, is somewhere between 15 and 18 lac…and I think, spending 12 lakhs or 15 lacs in a duration of 3–4 months is very big, huge…10 times the average salary of middle-class person…’* [1] (INR 14–15 lacs = approx. USD 17,000–18,300 or GBP 13,000–14,300).

*‘…there is limited income in the family. There is no end to treatment expenses for cancer…household is affected a lot…’* [1]*.*

The above excerpts are extracted from an exploratory study conducted to understand the treatment-related financial toxicities among head and neck cancer patients in India [1]. Financial toxicity (FT) intertwines with conflicting emotions and a pervasive sense of role dissatisfaction, compounding powerlessness, hopelessness and disrupted family dynamics, leading to profound feelings of loneliness, isolation and abandonment that intensify its burden [1–7]. In India, the projected number of cancer cases will rise from 1.46 million in 2022 to 1.57 million in 2025, with lung and breast cancers being the most prevalent types. The age-standardised incidence rate increases with age, particularly in the female reproductive age group (15–49 years) [8].

FT is a global issue that presents a pronounced challenge in low- and middle-income countries (LMICs) like India due to insufficient government investment in public healthcare, limited insurance coverage and substantial out-of-pocket expenditure (OOPE) [9, 10]. Low government health expenditure limits the capacity and quality of public sector healthcare, compelling over two-thirds of individuals to opt for the costlier private sector [9, 11]. Private healthcare provides the majority of both outpatient (70%–80%) and inpatient (60%) care. Nevertheless, data from the 2017–18 National Sample Survey revealed that merely 14% of rural and 19% of urban populations in India were covered by health insurance [9, 11].

Despite ongoing efforts, the current allocation of public funds towards healthcare in India remains below the recommended benchmark, with less than 2% of the GDP allocated, projected to reach 2.5% by 2025, still falling short of the WHO’s recommended 5% mark [12]. The National Institution for Transforming India (NITI) Aayog, a policy think tank of the Indian government, also addresses another pressing issue known as the: ‘Health Insurance for the ‘missing middle’ in India’. This highlights a significant segment of the population, spanning all income quintiles in both urban and rural areas, that lacks any financial protection by way of health insurance, despite having the means to afford it. The ‘missing middle’ represents a broad category that falls between the economically disadvantaged (who may receive government-subsidized insurance) and the affluent sector. This group is not financially challenged enough to qualify for government-supported insurance, yet they cannot afford high-value, comprehensive private insurance typically targeted at high-income individuals. This constitutes at least 30% of the population, which is approximately 40 crore (400 million) individuals. The actual number of uncovered individuals is likely even higher [13]. Furthermore, a significant portion of individuals covered by other public or private insurance schemes often lack comprehensive coverage. This results in ineffective and inadequate protection, leaving them with limited financial support for healthcare expenses [14–19].

With over three fourth of cancer treatment expenses being covered directly by patients through OOP spending, this situation leads to catastrophic healthcare expenditures and distress financing for hospitalisations, affecting both rural and urban populations in India [9, 10, 20–22]. Studies from high-income countries like United states, Italy and Japan show that public health insurance or low-cost treatment system or government-funded hospitals may not always provide protection against FT in individual cancer patients [23]. It is thus crucial to highlight here that, unlike countries with good public health insurance and fixed OOPE cost models, financial planning plays a significant role in treatment planning and goal-setting in LMICs like India, where patients bear either the entire treatment costs or a major share of it [23].

Thus, addressing the issue of FT in cancer care right at the diagnosis, when palliative care is integrated into the care continuum, may be helpful in improving the planning and management of healthcare expenses. This necessitates awareness of healthcare expenditures, expanded insurance coverage, increasing financial literacy and the implementation of robust financial planning measures to mitigate adverse financial consequences, especially in the last 6 months of life, where a lack of uniform palliative care facilities in the cancer institutions across the country raises the burden exponentially due to repeated hospital admissions [6, 10, 23–30, 31–33].

There is a dearth of literature about FT related to cancer treatment in Indian settings [10, 34]. In this article, we aim at: a) reviewing the available literature about FT as a source of suffering and the current propositions to its management in an Indian context; b) exploring potential solutions and proposing a possible framework for preventing and managing FT in patients undergoing cancer treatment; c) highlighting the benefits of early integration of palliative care and a timely transition to hospice in terms of preventing FT in the last months of life.

## Investigating the contributing factors and decodi﻿ng the impact of FT

Given the variation in the healthcare financing s﻿ystems globally, we did a literature search in PubMed to understand the available data for the financial burden of cancer in different cancer care settings in India using the following keywords: ‘financial stress’, ‘cost of illness’, ‘health expenditures’, ‘neoplasms’, ‘India’, ‘neoplasms, economics’ and ‘insurance, health’. The available literature primarily focuses on determining the prevalence of FT, OOPE, and the sources and nature of catastrophic health expenditure (CHE) faced by cancer patients and their families [3, 10, 21, 34–40, 41, 42].

The prevalence of FT varies across various healthcare settings, ranging from 30% to 90% [10, 34–38]. Similarly, OOPE and CHE vary among different study settings and various socio-demographic characteristics of cancer patient populations [3, 10, 21]. Overall, the available data indicates that expenditure is influenced by factors such as socio-economic status [3, 35, 36], patient age group [10, 36, 42], regional distribution [10, 21], availability of insurance schemes and coverages there of [3, 34], cancer type and stage [10, 39], treatment options [3, 10, 37] and healthcare settings [3, 10, 21, 37, 42].

Navigating through the journey of cancer treatment is likened to a war-like situation, causing severe blows to individuals and their families, significant socioeconomic disruption, and the risk of nonadherence to the treatment and its fatal consequences [39, 40]. The impact of cancer diagnosis and treatment can be better understood in two phases, i.e., the immediate and the delayed impact.

Immediate impact: The initial high costs associated with diagnosis, opinions, multiple investigations and navigating the complex healthcare system to find the right place for treatment put immense pressure on financial capabilities within a short timeframe, similar to initial surgical strikes [1, 3, 34, 37, 39]. Most of the cancer care facilities in India are concentrated in urban areas [21, 43]. Seeking treatment away from home brings the challenge of managing travel and multiple accommodations. Financial burden forces people to sacrifice their leisure activities, opting for cheaper but inconvenient transportation (risking infection) and limiting joyful moments with family, leading to guilt [3, 10, 21, 40].

Delayed impact: The devastating impact of this financial war extends beyond what meets the eye. As treatment proceeds, gathering funds continually becomes an arduous task. The loss of a job or inability to work causes a loss of income for patients and family members [39]. This is compounded by non-medical expenses for the wider family, including travel, lodging, food and visitor costs [3, 34, 41] High expenditures, depleting financial reserves and the need to bridge this financial gap with personal funds lead to the utilisation of savings, asset sales, borrowing, the accumulation of debt and the burden of loans, leading to medical bankruptcy [21, 39, 40]. The complex interplay between cancer treatment, supportive healthcare for the patient and financial challenges like family expenditure, including marriages, family spending, mortgage payments, job loss, child education and employability concerns adds to worries [1, 3, 10, 31, 44]. Financial dependence poses a threat to moral identity [1, 10, 45]. Loss of social roles, lack of support and potential abandonment exacerbate challenges. Financial distress strains relationships and has profound psychological effects [3, 10, 36, 45].

The proposed solutions in the available literature for FT focus mainly on seeking government support, cost reduction through generic medications, improving cancer care in public hospitals, and the utilisation and improvisation of health insurance schemes [3, 10, 34, 35]. But these solutions may come with other unforeseen challenges like cumbersome processes in seeking health insurance benefits, distrust in insurance companies, uncertainties surrounding claims and government reimbursements, inadequate quality of healthcare in government-funded hospitals, quality concerns with generic medicines and commercialisation in private healthcare [1, 3, 10, 21, 40].

In response to a recent query in the parliament of India, it was responded that India’s health insurance segment contributed 0.34% points out of the overall 1% point of general insurance (including health insurance) penetration in the country [46]. The National Health Accounts estimates for 2019–20, published by the National Health Systems Resource Centre, Ministry of Health and Family Welfare, India, estimate that the total OOPE burden in India for healthcare services is over 300 billion INR (Indian Rupees) [47]. The ‘traditional savings priorities in India’ focused on saving for buying a housing, education and marriage, with a preference for accident and life insurance rather than healthcare insurance, which contributes to the inability to cover escalating cancer care expenses, particularly for individuals who are currently in their forties and beyond [1, 21, 38, 48]. There is insufficient emphasis on enhancing financial literacy, including insurance literacy, planning and awareness among healthcare professionals, patients and their families in India [10, 49, 50]. Improving these aspects prevents ill-informed financial decisions, that may help mitigate the downstream consequences of financial hardship, such as poor treatment adherence and reduced emotional well-being [50–56]. Financial planning measures like optimising insurance, budgeting, cashflow optimisation, categorising assets, creating contingency funds and leveraging assets to create temporary liquidity form an important part of overall ‘financial literacy’ (in addition to ‘insurance literacy’) in order to address crisis phases during the disease process, but sadly this remains unexplored. There is a growing need for financial literacy among health professionals in the country [56]. Low financial literacy and consequent lack of confidence prevent the participation of healthcare professionals in cost-related discussions with patients and their families [57].

## Exploring strategies for future preparedness

### Importance of early planning upon cancer diagnosis: mitigating the burden of treatment expenses- when and where to begin?

Addressing the fear of cost burdens right at the time of diagnosis, along with early and strategic financial planning as described in the framework mentioned below, by engaging a multidisciplinary team comprising professionals like oncologists, palliative care team, social workers, counselors and financial planners or navigators sensitised to cancer care, can help in preparing for the upcoming expenses and treatment planning [10, 23, 57–61]. Building a support network with family members helps collectively navigate the challenges of treatment expenditures, allowing patients and their families to prioritise their well-being, reducing the stress of payments and paperwork [1, 59, 62].

### Tracking and analyzing expenses, consolidating assets and creating liquidity

The existing literature in the Indian context lacks a comprehensive insight into the specific frameworks or processes necessary for an individual’s readiness for health-related situations. Relying solely on health insurance may no longer be adequate to shield patients and their families from financial hardship and its associated adverse consequences in a country like ours with low insurance coverage and high OOPE [23, 60, 63]. Rather, in a survey of 404 cancer patients, it was concluded that a high level of ‘financial literacy’ (includes knowledge, skills and behaviours for making well-informed financial decisions) helps in mitigating the adverse outcome of lower health insurance literacy, in patients with cancer [51]. Thus, we explored upon a systematic approach, as mentioned above, for individual's readiness, based on the available global and regional literature [23, 25, 62, 64–68].

#### Tracking expenses and organising data

The family’s response to a cancer diagnosis involves accepting the battle ahead and assessing the financial situation by evaluating current cash flow (income and expenses), investments, assets and liabilities [69].

Gathering all necessary documents, whether paper-based or digital, is the initial step [64, 69, 70]. For organised individuals, the process can be straightforward, while others can begin with their bank account statements. By reviewing transactions and investigating debit entries, past investments can be identified, leading to the swift compilation of an asset list.

**Figure 1.**
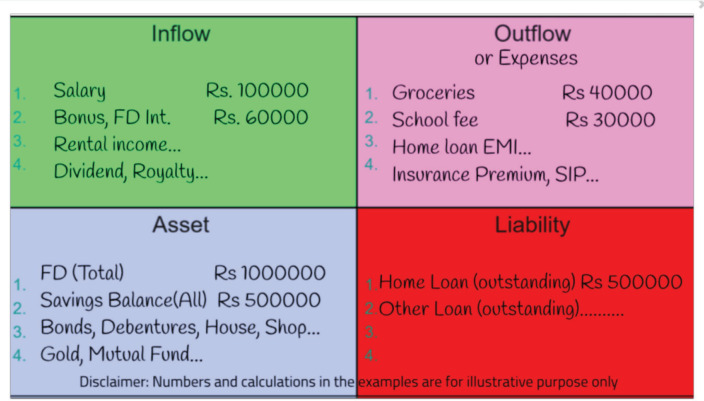
Four-quadrant format to record income, expenses, assets and liabilities (adapted with permission from https://ascentcapitalonline.com/ and https://hospiceindia.com/financial-aspects-of-care).

Tracking expenses is the next crucial step. Utilising a user-friendly four-quadrant format for recording income, expenses, assets and liabilities is recommended (Figure 1) [64, 65, 70, 71]. This organised data becomes a valuable asset and facilitates informed decision-making during the challenges ahead [64, 70, 71].

To complete the quadrant, the user should fill in one section at a time with relevant information from bank statements or other documents. In the ‘Inflow’ section, individuals must record incoming money from various sources, such as salary, interest (e.g., from fixed deposits), rental income, commission income or royalties. Seeking assistance from chartered accountants or financial planners can ensure accuracy in this quadrant. The ‘Outflow’ section pertains to the outward movement of money, representing expenses and where the money is spent. This involves listing down expenditures such as groceries, school fees, loan payments (e.g., home loan and car loan equated monthly installment (EMIs)), utility bills, salaries for employees and domestic help, credit card payments and so on [62, 64, 70–72].

In the ‘Assets,’ individuals must create a list of all movable and immovable investments. These assets include items from which ‘interest’ is received or where ‘ownership’ is held, such as fixed deposits, savings account balances, bonds, debentures, properties, gold and more. The fourth quadrant, labelled ‘Liabilities,’ requires individuals to list all types of loans, such as home loans, personal loans, credit card loans, gold loans and others. This comprehensive personal data tracking method helps individuals gain a clear overview of their financial situation and make well-informed decisions [62, 64, 70–72].

#### Classifying, consolidating and utilising assets

After completing the above exercise, the ‘assets’ can be categorised into three groups: liquid, semi-liquid and illiquid. Liquid assets can be quickly converted into cash within a short period of time [73, 74]. Semi-liquid investments require some time before they can be encashed or partially liquidated. Illiquid investments involve assets that are pending acquisition, require significant efforts or are currently non-liquidatable [64, 70, 74, 75].

The next step is to: consolidate assets(both movable and immovable), generate temporary liquidity through loans and overdrafts to prevent running out of money, and complete essential paperwork for the crisis situations and estate planning [64, 65, 70, 74, 76]. When confronted with health challenges, households with limited financial assets experience a heightened demand for borrowing but are also more likely to face liquidity constraints [77].

#### Creating temporary liquidity: understanding loans, loans against property and overdrafts

Liquidity is important throughout cancer care, [74, 75, 78] especially if timely transitioning to hospice care is lacking. Studies show that costs of care may increase exponentially at the end of life, primarily driven by inpatient care and the urgency with which it is approached [79–81].

For assets that become illiquid due to no or low demand, temporary liquidity can be generated by obtaining a loan against the property from banks or non-banking financial companies, based on a valuation report [23, 64, 65, 69, 70]. To understand a loan against property and an overdraft, let us first try to understand a loan. The distinction between a loan and an overdraft lies in their characteristics. A ‘loan’ typically has a fixed tenure, interest rate and EMI. When obtaining a loan against property, the property serves as collateral, and the bank or financial institution holds the original documents until the loan is fully repaid. On the other hand, in an ‘overdraft’, interest payments are based on the actual amount used. It allows account holders to withdraw funds exceeding their bank account balance up to the limit approved by the bank [64, 66, 70, 82, 83].

An effective management strategy for financial hardships and urgencies can shield families from the detrimental impacts of FT by tiding over the crisis phases. Managing ‘idle funds’ parked in regular savings accounts and utilising the same to generate higher returns by using short-term debt instruments can help reduce the negative impact [64, 65, 70, 84].

### Streamlining paperwork and understanding banking operations for effective financial management

People fighting cancer may face difficulties signing documents or carrying out paperwork due to the disease, hospitalisation or adverse effects of cancer treatment. To address these challenges, pre-planned and authorised paperwork becomes essential. This ensures important matters are taken care of efficiently, even when patients cannot handle their paperwork personally [64, 65, 69, 70].

Understanding banking operations includes knowledge about various types of account holdings, modes of operation within bank accounts, nomination processes and other relevant banking procedures [64, 70].

It is vital to comprehend legal instruments such as power of attorney, nominations and the selection of suitable estate planning tools. A ‘Power of Attorney’ is granted to a trusted individual, authorising them to conduct general or specific transactions on behalf of the patient. A ‘Nominee’ is supposed to take care of the assets of the deceased and ensure the transfer of these assets to the declared beneficiaries. A nominee does not possess legal ‘ownership’ automatically, only by virtue of being a nominee.

Overcoming familial beliefs or stigmas related to ‘estate planning’ and writing a ‘will’ is essential. It is also crucial to understand the distinctions between a ‘nominee’ and a ‘successor’, along with the rights linked to ‘ownership’ and their transfer. Implementing these strategies reduces stress and enables greater focus on treatment and well-being during cancer care [64, 66, 69, 70].

### Estate planning

An ‘Estate plan’ covers the division of finances and assets and the passing down of family heirlooms or other keepsakes. Estate planning is a process that requires time, consultation with the family and the patient’s comfort [33, 62, 69, 70]. Within our society, there is a prevailing taboo about writing a will, and estate planning, resulting in a tendency to avoid discussions on topics like estate planning [85, 86]. Despite societal taboos, addressing topics like wills and trusts is essential to acknowledging life's uncertainty. Waiting until 50–60 years to write a will is misguided; rather, healthy individuals who are earning and owning assets should consider writing a will or exploring alternative estate planning tools with legal consultants, regardless of their age [33, 69, 70, 87].

An individual who have diligently accumulated assets such as property, money and more throughout their entire life but fails to adequately address the seamless transfer of these assets to their chosen legal successor will lose peace of mind as they near the end of their lives, and this can become a cause of psychosocial distress [69, 87]. It is crucial to recognise its significance in palliative and hospice care.

### Understanding health insurance

Improved health insurance literacy is linked to reduced financial hardship [51]. Additionally, as discussed earlier, enhanced ‘overall financial literacy’, leading to sound financial decision-making and greater financial capability, can help alleviate the negative effects of low health insurance literacy on financial hardship [51]. Review of health insurance with emphasis on three crucial points to consider when evaluating any insurance policy:

- Coverage: What does the policy encompass? Understanding the policy’s included medical services and treatments to match individual healthcare needs.
- Exemptions: What expenses are exempt or not covered? Identifying non-covered expenses to plan for potential out-of-pocket costs.
- Exceptions: What is covered but only under specific circumstances? Recognising covered items under specific circumstances to avoid surprises when accessing healthcare services.

By assessing these aspects, individuals can select a suitable health insurance policy that meets their healthcare needs and financial considerations [65, 70].

## Early access to palliative and supportive care and financial well-being

Accessing high-quality palliative care is crucial for addressing FT and enhancing the overall quality of life and overall satisfaction for cancer patients and their families. Literature, including systematic reviews, shows that the timely introduction of palliative care is associated with a lower financial burden compared to routine care. This advantage stems from reduced healthcare utilisation, including fewer hospital admissions, emergency room visits, outpatient facilities, shorter hospital stays, lower visitor costs and lesser admissions to hospital and timely transition to the hospice care [10, 88–93] However, up to 38% of patients receive chemotherapy or life-sustaining support in the last month of life, while the majority (up to 66%) do not receive palliative or hospice care [94]. To address this, timely recognition of the futility of treatment and expanding the use of palliative care, hospice and home-care services earlier in the disease course are recommended [79–81, 94, 95]. A landmark study by Temel *et al* [92] reported that patients with non-small cell lung cancer who received both standard oncologic care and early palliative care experienced notable enhancements in their quality of life, including their emotional well-being, as well as longer survival rates [92]. Similarly, a prospective, randomised controlled trial done for palliative care patients with advanced-stage COPD, congestive heart failure and cancer, in a home setting reported a statistically significant improvement in patient satisfaction scores, reduction in emergency room visits and hospitalisations, along with a substantial (45%) decrease in the overall cost of care, translating to a 33% reduction in health expenditure per patient [95]. Furthermore, as per our knowledge, there are no insurance plans that cover hospice care and palliative care health expenditures in India.

## Community support and resources

Addressing cancer-related FT requires a comprehensive approach involving patients, providers, communities and system or policy-level strategies [9, 10, 58, 89]. Patients can empower themselves through reliable information, second opinions and involving counsellors, social workers and financial experts familiar with cancer care [10, 24, 68, 96]. Advance care planning, documenting advance directives and stating care preferences, reduces cost burden especially towards the end of life [97, 98] Exploring government health schemes offers financial support during treatment [10, 43, 58]. Healthcare providers play a crucial role through active early screening for FT and open communication with patients and families [10]. Collaborating with financial counsellors and planners, social workers, following evidence-based guidelines and early palliative care integration are vital strategies [10, 58, 89, 90, 92, 99, 100].

At the community level, raising cancer prevention awareness, promoting early diagnosis through screening, involving com﻿munity care teams in cancer care, integrating palliative care and home care programs and improving financial literacy is crucial [10, 24, 37, 51, 68, 95]. Financial support from groups, like non-profit organisations, and cancer foundations can help alleviate burdens. Crowdfunding and social media campaigns are some of the effective fundraising tools [101–103].

Organisational, state or policy-level interventions are vital. Essential measures include increasing public health spending, promoting government health schemes, standardising and quality-checking generic pharmaceutical products, early screening for FT and integrating cost discussions into medical training. Additionally, a patient and caregiver financial literacy program in the context of cancer care in India, whether conducted in person or online, is essential [1, 10, 23, 24, 37, 51, 68, 104]. Organisational or state-level collaboration with financial counsellors or financial planners who are aware and sensitised to the Indian healthcare system, needs to be incorporated into cancer care early in the disease trajectory [23, 51, 68, 105]. Sensible reimbursement policies, preventive measures, health technology, early integration of palliative care, palliative care education and training, strengthening institutional and home care support for palliative and hospice care are crucial steps. Medical education should focus on decision-making, assessing futility, suffering, person-centric care and end-of-life care policies at institutional levels [10, 23, 93, 106–108].

Some of the currently available financial support schemes in India are [12, 16, 67]:

1. Rashtriya Arogya Nidhi (RAN) consists of three components: RAN for non-cancer life-threatening diseases, Health Minister’s Cancer Patients Fund for cancer patients and a scheme for financial assistance up to INR 2 million for specified rare diseases.
2. Health Minister’s Discretionary Grant
3. Pradhan Mantri Jan Arogya Yojana provides cashless coverage of up to INR 0.5 million per eligible family per year for listed secondary and tertiary care conditions.
4. The ESI Scheme
5. Central Government Health Scheme
6. Prime Minister relief fund
7. Chief minister relief fund.
8. The Indian Cancer Society-Cancer Cure Fund
9. Other regional and state government schemes like Swasthyasathi scheme in Kolkata, India
10. Income tax rebate: Under Section 80D and 80DDB and others should be explored.
11. Travel concessions by air and railway, subsidised or free accommodation near hospitals by NGOs, free food and nutrition support from various organisations, financial aid from pharmaceutical and non-pharmaceutical companies and free cancer treatment at select government hospitals. www.cancerassist.in serves as a comprehensive assistance resource for cancer patients.

## Conclusion

Addressing the burden of FT in cancer care for a population with high OOPE and CHE, low or no insurance coverage for a majority of people and inadequate government expenditure in the public healthcare sector requires a comprehensive strategy encompassing financial preparedness, health insurance navigation, early financial counselling and planning, enrolment in the eligible government schemes, early integration of palliative care and timely transition to hospice care, advanced care planning, documenting advance directives, end-of-life care policies and estate planning. Well-planned finances, coupled with organised financial data management, can prevent families from slipping into FT. Utilising community support, non-profit organisations and cancer foundations is crucial, along with advocating for increased awareness and policy changes. Early proactive measures, seeking professional guidance and leveraging available resources can help mitigate the financial impact of cancer. Education and awareness are especially crucial in rural areas, where government infrastructure and engagement with educated individuals can disseminate knowledge about healthcare funding options and support mechanisms. Innovative approaches, such as low-interest loans and crowdfunding platforms, can aid in managing the financial challenges of cancer care. Although this article depicts the impact of cancer-related FT and its possible mitigating solutions in the Indian context, they can be extrapolated to other LMIC settings globally. There is a pressing global need for increased research and education covering financial literacy, awareness and planning to effectively manage various aspects of FT in cancer care.

## Conflicts of interest

No conflict of interest.

## Funding

No funding received.
